# Quality Parameters of PE–Pomace Based Membranes

**DOI:** 10.3390/membranes12111086

**Published:** 2022-11-01

**Authors:** Izabela Betlej, Renata Salerno-Kochan, Piotr Borysiuk, Piotr Boruszewski, Sławomir Monder, Krzysztof Krajewski, Bogusław Andres, Barbara Krochmal-Marczak, Elżbieta Pisulewska, Leszek Danecki, Stanisław Pochwała

**Affiliations:** 1 Institute of Wood Sciences and Furniture, Warsaw University of Life Science—SGGW, 159 Nowoursynowska St., 02-776 Warsaw, Poland; 2 Institute of Quality Sciences and Product Management, Krakow University of Economics, 27 Rakowicka St., 31-510 Krakow, Poland; 3 Department of Plant Production and Food Safety, Carpathian State College in Krosno, 12 Dmochowskiego St., 38-400 Krosno, Poland; 4 Research and Development Centre for Wood-Based Panels, 10a Adama Mickiewicza St., 83-262 Czarna Woda, Poland; 5 DELTA, 16/10 Kuźnicy Kołłątajowskiej St., 31-234 Krakow, Poland

**Keywords:** HDPE, plant-based membranes, waste management, quality parameters, water vapor permeability, contact angle, shrinkage

## Abstract

This paper presents the results of research on selected mechanical and physical properties of polyethylene membranes containing 50% of the plant fraction obtained as waste from an edible oil press. The produced biomembranes were characterized by low tensile strength (2.02–4.28 MPa). The addition of plant material will not adversely affect the barrier properties such as water vapor permeability or the contact angle. Additionally, there was a discoloration of the characteristics affecting the shrinkage of the membrane. The presence of the plant component clearly lowered the shrinkage of the material. This research is important and provides valuable knowledge on the possibilities of using plant waste and the direction of the potential application of the materials produced with their use.

## 1. Introduction

The development of agriculture and the food industry is associated with the creation of more and more new products, but also various types of post-production residues. Unused by-products can be a problem for the industrial plants where they are generated, but also imply a potential environmental hazard. A separate issue is the cost of their storage and disposal, which is closely related to the protection of the natural environment. Therefore, appropriate waste management, carried out in accordance with the concept of sustainable development, based on the principles of the circular economy, is extremely important [1]. It can bring a number of economic, social and environmental benefits, including reducing the costs of exporting and disposing of waste that can be used as a raw material for the production of new products [2,3]. The best and fully ecological treatment of waste is to prevent its formation. The re-use of by-products, previously regarded as processing residues, may also significantly reduce the negative effects of their impact on the environment.

Oil production processes generate significant amounts of by-product in the form of expeller. Depending on the capacity of the equipment, 100 kg of raw material used for the production of oil produces from 20 to 70 kg of pomace with high utility potential [4]. Such a large amount prompts the search for methods of their management. Currently, bagasse is used, among others as a high-protein feed for farm animals [5], as a biofuel [6] and as an organic fertilizer [7]. It seems that there is also the possibility of using them for the production of a new type of bioplastic with various applications.

In the literature on the subject, there are examples of the production of bioplastics such as films or membranes from synthetic biodegradable polymers. However, their production is expensive, so in order to reduce their production costs or improve their physical properties, they are combined with natural polymers such as polysaccharides (e.g., chitosan) [8] or plant proteins (e.g., soybean) [9]. Protein or polysaccharide membranes are a good barrier against the access of oxygen and carbon dioxide, which is important from the point of view of the use of such bioplastics for the storage of food products [10]. However, this type of bioplastic is characterized by a low elongation of a few percent, which significantly limits their application [11]. Compared to traditional plastics made from fossil sources, bioplastics based on plant ingredients have a number of valuable advantages. First of all, they allow the saving of raw materials thanks to the use of cyclically renewing biomass. In addition, their production and use are carbon neutral, which means that their processing does not contribute to the increase in the production of carbon dioxide. Moreover, some types of bioplastics are biodegradable [12]. On the other hand, bioplastics containing an additive of plant origin may exhibit bactericidal and fungicidal properties [13,14]. Fiorentini et al. [15] showed that biopolymers based on plant pectins inhibit the growth of certain pathogens, such as *Escherichia coli*, *Staphylococcus aureus* or *Candida albicans*.

The qualitative characteristics of membranes based on polymers with the addition of plant ingredients are crucial in terms of their potential application. The plant additive may influence the mechanical properties, stiffness, light transmittance, gas barrier, antimicrobial properties [16,17] or dielectric properties [18]. Membranes with low gas or water vapor permeability are believed to be important in the preservation of certain food products [19]. Šimkovic et al. [20] showed that sugar beet holocellulose can be used in the production of a composite film with high strength and stiffness. Santhosh et al. [14] give examples of the strength properties of films and coatings based on plant ingredients, such as mango, grape, potato and many others, showing very different results of tensile strength and elongation. Deng and Zhao [21] showed that the tensile strength of polyethylene (PE) film with additives based on grape pomace extracts ranges from 1.12 to 4.04 MPa. The same authors also demonstrated poor water barrier properties as a result of the presence of an ingredient with hygroscopic properties. The permeability of water vapor and gases is undoubtedly an important feature that determines the potential use of bioplastics. Jafarzadeh et al. [22] showed that plant components (especially of protein origin) present in membranes, increase water vapor permeability, but this process can be prevented by adding an inorganic component.

Additionally, the application of nanotechnology allows for the production of biodegradable nanobiopolymers with unique mechanical, thermal and antimicrobial properties [23,24,25,26]. Sarwar et al. [18] showed that cellulose-based nanocomposite materials, modified by the introduction of Ag ions, effectively inhibited the growth of MRSA bacteria, which are harmful to health, and that they did not affect the cytotoxicity of cells, which means that they can be described as biocompatible.

The aim of the presented research was to characterize selected physical and mechanical properties of polyethylene membranes modified with plant-derived ingredients obtained as waste in the production of cold-pressed oils. The plant particles were not modified, which lead to the development of a cheaper process for the production of biomaterial with specific properties and the need to check the production possibilities. The results of this research allowed the determination of the quality characteristics of the manufactured membranes and definition of the directions of their potential use.

## 2. Materials and Methods

The material for the production of the membrane was lignocellulosic material in the form of pomace after oil extraction (black cumin, flax, corn) (AL-PHADAR, Nienaszów, Poland) and high-density polyethylene-HDPE (Hostalen GD 7255, Basell Orlen Polyolefins Sp. z.o. o., Płock, Poland).

### 2.1. The Process of Producing Membranes

In the first stage, granules were produced from thermoplastics and pomace after oil extraction of black cumin, flax and corn seeds. The pomace was preliminarily milled to a dusty fraction (<0.300 µm) using a knife grinder DS-2/OBR (OB-RPPD Sp. z o. o., Czarna Woda, Poland). The fragmented pomace was combined with thermoplastic particles with the assumed mass fraction of 50% and homogenized in a high-speed mixer (KMOD SGGW, Warszawa, Poland). The composite was then obtained using an extruder (Leistritz Extrusionstechnik GmbH, Nürnberg, Germany). The temperatures in the individual sections of the extruder were 170–180 °C. After cooling, the composite was ground using a DS-2/OBR knife grinder (OB-RPPD sp.z o. o., Czarna Woda, Poland) to the form of a fine fraction, which was used for further processing to the form of a membrane of a specific thickness.

The ground composite was divided into two fractions with different particle sizes (particles fraction above 1.5 mm and particles fraction below 1.5 mm). For further use, the fraction with particle size below 1.5 mm was used. 5 g of the ground composite (with predetermined particles size) was placed between heat-resistant films. A weight was placed centrally on the lower heat-resistant foil. The upper part of the sample was covered with the upper heat-resistant foil. Between the lower and the upper foil there were aluminum spacers about 0.32 mm thick. Additionally, the lower and upper heat-resistant foil was covered with a layer made of aluminum. The thickness of each layer was 5 mm. The material was placed in the press in this form. The pressing process was carried out in a one stage press (AB AK Eriksson, Mariannelund, Sweden). The assumed thickness of the membranes was obtained with the usage of proper spacers.

Parameters of the pressing process: the pre-pressing, low-pressure stage (unit pressure during pre-pressing was from 0 MPa to 0.2 MPa), pressing time was 30 s, with the time measured from the double-sided contact of the mat with the upper and lower shelf of the press; the proper pressing, intermediate pressure stage, after 30 s of pre-pressing, the unit pressure was increased to the level of 1.5 Mpa and this pressure was maintained for 30 s; the final pressing, the stage of increased pressure, in the last stage of pressing, the unit pressure in the process is increased to the level of 2 Mpa and the pressing time was 30 s.

Hot pressing parameters: total time 90 s, pressing temperature 160 ± 5 °C.

Cold conditioning: temperature 20–40 ± 5 °C, the biomembranes were placed in a cold press immediately after being removed from the press for hardening; cold pressing was carried out at the unit pressure of 1 MPa for 180 s; cold pressing was conducted in using the press from Zakłady Urządzeń Przemysłowych, Nysa, Poland.

The control sample was a membrane made of pure polyethylene. The method of preparing the control sample was identical to the way in which the membranes with the addition of pomace after oil extraction were prepared.

Immediately after pressing, the membranes were in the form of circles, which were then cut into squares (from them subsequent forms were cut out, the dimensions of which were dedicated to specific tests).

The thickness of the membranes produced was measured using a thickness gauge—Ultrameter AB400 model (Metrison Sp.z o. o., Mościska, Poland). The measurement was performed in ten replicates.

To determine the apparent density of biomaterials, pieces with dimensions of 50 mm × 50 mm were cut, and then their volume and weight were determined. The weight of the cut pieces was determined using a precision balance model 1000.X2 (Radwag, Radom, Poland).

The humidity of the used ground composite and the produced biomaterial was determined by the dryer-weight method—WPS10S (Radwag, Radom, Poland).

The membranes obtained are marked with the following symbols: PE-B—membrane containing polyethylene and pomace after oil extraction of black cumin seeds; PE-F—membrane containing polyethylene and pomace after oil extraction of flax seeds; PE-C—membrane containing polyethylene and pomace after oil extraction of corn seeds.

**Figure 1:**
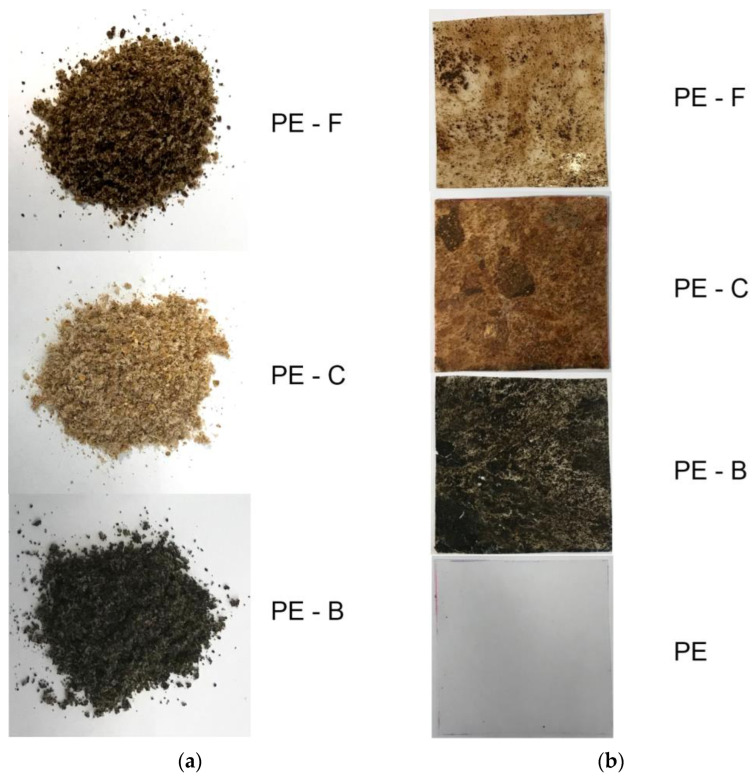
(**a**) PE–pomace plant-based ground composites; (**b**) PE–pomace plant-based membranes.

Visualization of ground composite used in the tests and membranes are shown in Figure 1a,b.

### 2.2. Quality Parameters

#### 2.2.1. Tensile Test

Tensile strength tests were carried out based on the guidelines of ISO 527-1. The tests were performed on a testing machine model Instron 5544 (Instron Ltd., High Wycombe, UK). The shape and dimensions of the samples were in accordance with the guidelines of ISO 527-3. The tensile tests were carried out at a head speed of 100 mm/min. Breaking load (N) and absolute elongation (mm) at break were measured. Additionally tensile strength value (MPa) and Young’s modulus (GPa) were calculated. The tests were performed in ten replicates.

#### 2.2.2. Water Vapor Transport

Water vapor permeability was determined by the cell method with the use of a Max 50 moisture analyzer (Radwag, Warsaw, Poland). The disc-shaped pads with a diameter of 54 ± 2 mm were placed on the surface of the perforated tray. A vessel with distilled water which was maintained at a temperature of 30 ± 2 °C was placed in the test apparatus. After 1 h of acclimatization of the samples under these conditions, the initial weight of the water vessel (m_1_) was determined. Then, after another 1 h of acclimatization of the samples under the same conditions, the weight of the vessel with water (m_2_) was determined again. The water vapor permeability value expressed in g/m^2^ · 24 h was calculated according to the Formula (1):

WVP = (m_1_ − m_2_) · S^−1^ · (24 · t^−1^) · 10^4^ [g · m^−2^ · 24 h] (1)

where: m_1_—mass of water in the moisture analyzer after 1 h [g], m_2_—mass of water in the moisture analyzerafter 2 h [g], S—surface area of the test sample (19.625 cm^2^), t—determination time [1 h], 10^4^—value resulting from the conversion of cm^2^ to m^2^.

#### 2.2.3. Determination of the Contact Angle

The contact angle measurement was performed with a Haas Phoenix 300 goniometer (Surface Electro Optics, Suwon City, South Korea). In the study, the angle between the tangent to the drop contour and the straight line passing through its base was determined. The image analysis system Image XP v. 5.8 (Surface Electro Optics, Suwon City, South Korea) was used to determine this measurement. The analysis of the change in wettability was performed after 5, 20, 40 and 60 s from the moment of depositing a drop of distilled water on the surface of the membrane. Measurements were made in ten replicates, at 50% air humidity and at a temperature of 21 ± 2 °C.

#### 2.2.4. Determination of Shrinkage

The membranes shrinkage test was performed using a Labthink RSY-R2 shrink tester (Labthink International, Inc., Medford, OR, USA). The shrinkage test was carried out in distilled water, at a temperature of 100 °C, for 300 and 1800 s. The test specimens had the shape of circles with a diameter of 30.34 ± 0.01 mm. After the test, the diameter of the samples was measured in two perpendicular directions. The contractility was measured in ten replicates. The shrinkage was determined as a percentage.

#### 2.2.5. Measurement of Pomace after Oil Extraction Particles’ Size and Their Percentage Share in the Membrane’s Surface

Assessment of the share of pomace after oil extraction particles in membrane’s surface was performed using the Delta Optilac Evolution 100 light microscope (Delta Optical Sp. Z o. o., Warsaw, Poland), using a 10× magnification, equipped with a Levenhuk M1000Plus camera (Levenhuk Poland Sp. Z o. o., Warsaw, Poland). The particle size was measured in at least two opposite directions, using the DLTCamViewer program.

**Figure 2:**
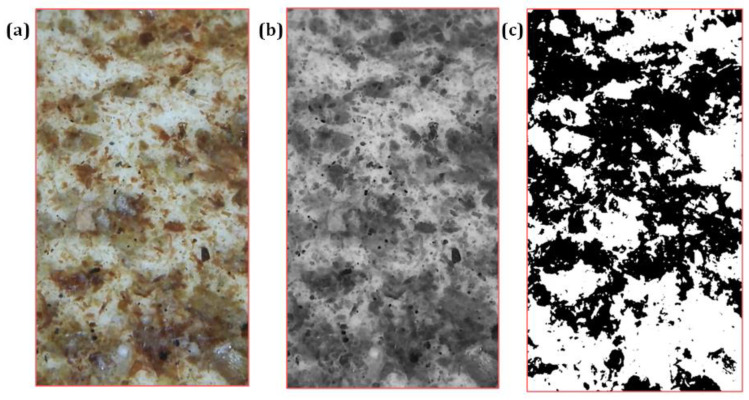
Successive stages of transforming material photos: (**a**) microscopic photo, (**b**) photo after conversion to 8-bit form, (**c**) photo after conversion to binary form.

The percentage share of pomace particles in 10 mm^2^ of the membrane’s surface was determined with ImageJ2 image analysis software (Fiji v.1.52i). The study used a Delta Optical Smart 5 MP PRO microscope (Delta Optical Sp. Z o. o., Warsaw, Poland), integrated with the Delta Optical Smart Analysis Pro software. The percentage share of pomace particles in the membrane’s surface was determined with an accuracy of 5%. The microscopic photos of the material samples were digitally processed by pre-equalizing the brightness level of the photos and then converting to 8-bit black and white image and converting to binary form. To determine the percentage share of organic material particles on the surface of the tested samples, the option “Analyze particles” was used, available in the above-mentioned section. Below are the steps of digital photo transformation on the example of one of the samples containing the addition of flax particles (Figure 2).

### 2.3. Statistical Analysis

Statistical analysis of the results was carried out using Statistica version 13 (TIBCO Software Inc., Palo Alto, CA, USA). The analysis of variance (ANOVA) was used to test (α = 0.05) for significant differences between variants. A comparison of the means was performed using Tukey’s test, with α = 0.05.

## 3. Results and Discussion

Mechanical properties of materials are among the most important quality parameters of materials. Among them, the tensile strength is of particular importance, which is the basic criterion in assessing the quality and usability of polymers. The addition of pomace after oil extraction particles usually lowers this property, therefore research on the improvement of modification methods that allow obtained bioplastics based on plant ingredients to possess appropriate strength properties is a difficult challenge.

**Figure 3:**
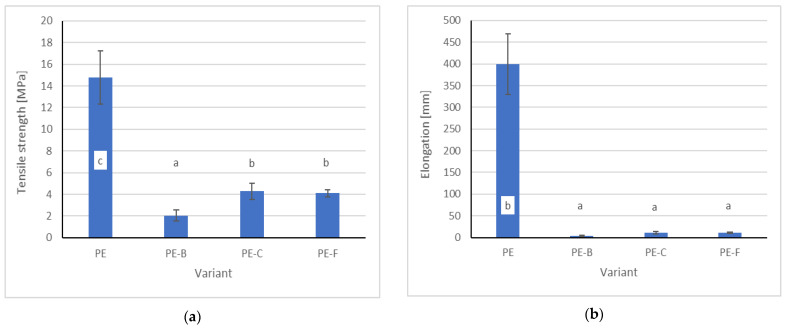
Mechanical properties of PE–pomace based membranes (**a**) tensile strength; (**b**) elongation; ^abc^ is the homogeneous groups by the Tukey test (α = 0.05).

Figure 3 shows the results of measurements of the mechanical properties of the tested membranes. On the basis of the obtained results, it was found that the addition of plant material in the amount of 50% significantly reduces the tensile strength and elongation of the sample as compared to pure synthetic polymer. The obtained results confirm the results of other researchers [27]. Santhosh et al. [14] indicated that plant components, such as polyphenols, significantly reduce the mechanical strength of some types of polymers, which is related to the interaction of phenolic compounds with water molecules. It should also be added that the black cumin seeds contain large amounts of polyphenolic compounds [28]. However, the main factor that reduces the mechanical strength is the reduction of the cohesive force between the PE particles by the addition of plant material. A large addition of a plant component makes the structure of the polymer more porous, and the presence of fiber contributes to a reduction in strength properties [29]. In the case of oil plants, seeds contain many fatty substances, also waxes and pectins, which weaken the mechanical properties of biopolymers, therefore, a frequent procedure improving the quality of these types of bioplastics is the pre-treatment of the raw material used [30].

For polyethylene, a very low value of Young’s modulus was obtained, i.e., approx. 0.32 GPa. Membranes with additives have much higher values of this parameter. It can be concluded that the additives reduce the deformability. The highest increase in Young’s modulus occurred for the corn modified membrane (18.3 GPa), while the lowest for the membrane with flaxseed (Table 1).

Despite the low mechanical properties of membranes with the addition of ingredients of plant origin, significant differences were found for the parameters tested for individual types of membranes (Table 2). The maximum strength values of PE-C and PE-F membranes were more than twice as high as in the case of the membrane containing black cumin extrudate (PE-B), not less than four times lower than in the case of the pure polyethylene membrane (Table 1). Higher breaking load values for membranes with the addition of flax and corn extrudate may be related not only to the type of plant component, but probably also to the thickness of the membrane (Table 2), which was about 30% higher compared to the membrane containing black cumin.

**Figure 4:**
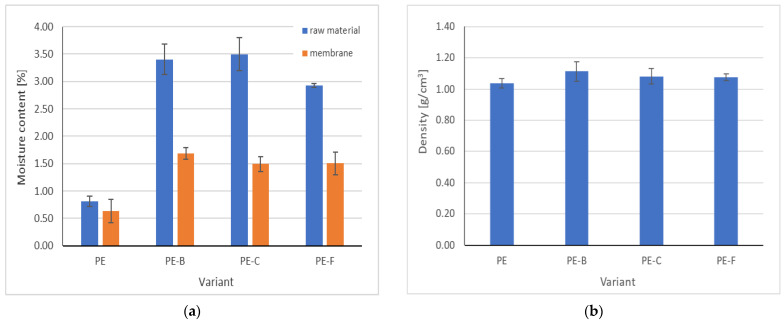
Physical properties of PE–pomace based membranes (**a**) moisture of raw materials and membranes; (**b**) apparent density.

Figure 4a shows the differences in humidity between the raw material used and the finished membrane. As can be seen, the fragmented raw material absorbs water more easily than the membrane made of it. The presence of the plant component influences the changes in humidity. There was no difference in the density of the produced PE–pomace plant-based membranes (Figure 4b).

**Figure 5:**
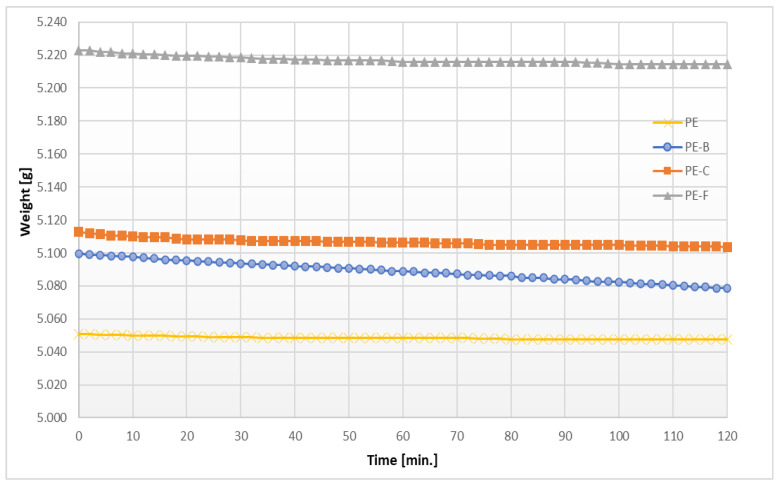
The course of mass measurement in the process of water vapor permeability determination on the example PE–pomace based membranes.

Another important parameter in the quality assessment of membranes is water vapor permeability. It determines the potential usability of the material. Research has shown that the presence of plant particles in the PE membrane causes slight changes in the properties of the obtained polymers towards the increase of water vapor permeability (Table 3). The water mass loss curves presented in Figure 5 as a result of its transport through the membrane during the measurement show that the tested membranes are characterized by low permeability, although this property changes depending on the type of plant components present in the membranes. Similar observations were presented by Jafarzadeh et al. [22]. The PE-B permeability is over four times higher than that of pure polyethylene and over two times higher than that of membranes with the addition of pomace after oil extraction of flax and corn seeds. The lower permeability of PE-C and PE-F in relation to the membrane with pomace after oil extraction of black cumin seeds may be related to the greater thickness of the membranes, however, the properties of the plant material and its chemical composition are probably the decisive factor [31], especially that the proportion of percent of plant particles per 10 mm^2^ of membrane’s surface is similar for all membrane types. Edhirej et al. [32] pointed out that the more hydroxyl groups in plant particles, the greater the water absorption of films with the addition of such particles. The key factor may also be the type of hydroxyl group donors, which may affect the degree of fusion with the synthetic polymer [33]. In turn, Escamilla–García et al. [34] found that the chemical groups of the components that make up the polymer affect the degree of interaction with each other, thus giving specific physical and mechanical parameters to the resulting polymers. It can therefore be assumed that the chemical composition of black cumin seeds affects the worse association between PE molecules, causing better water penetration within the membrane structure.

The tested membranes were characterized by low shrinkage at the temperature of 100 °C, and the addition of plant material additionally reduced the ability to change dimensions. The residence time of membranes at elevated temperature increased shrinkage (Table 3), no less significant differences were noted between membranes containing plant components. It can therefore be assumed that the plant additive introduced into the PE membrane reduces the internal tensions in the material, which is additionally confirmed by the research of other authors [35].

The measurement of the contact angle confirmed that there are statistically significant differences between pure polyethylene and a membrane containing a plant additive in the form of pomace after oil extraction of black cumin seeds (Table 4). The PE-B membrane showed better wettability compared to the membrane with the addition of pomace after oil extraction of flax and corn seeds, the wettability of which was similar (PE-F) or identical (PE-C) to pure polyethylene membranes. Thus, it can be concluded that the type of plant component in this case has a decisive influence on the change of the bioplastic wettability direction. Merino et al. [36] showed that good compatibility of avocado peel and seeds with the pectin polymer influences the results of wettability and water vapor permeability. In the research by Merino and Alvarez [36] it was found that the proportion of a given component in the polymer is of significant importance in determining the quality parameters of polymers based on plant components. A higher content of plant particles may lead to a more heterogeneous structure with the formation of aggregates that reduce the physical and mechanical properties of materials.

The results of the measurement of the contact angle showed a slight decrease in time, while for PE-B the change in the contact angle after 60 s after the drop of water was applied to the membrane was the highest (Table 4).

**Figure 6:**
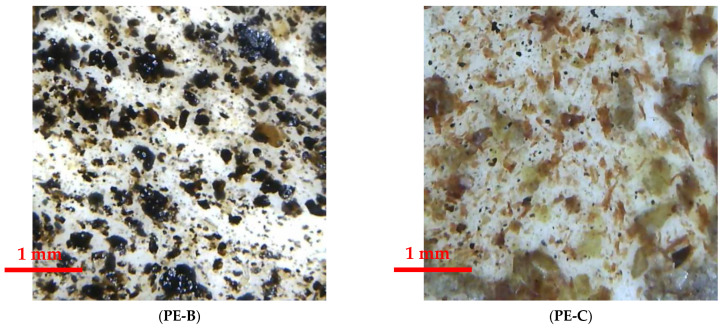

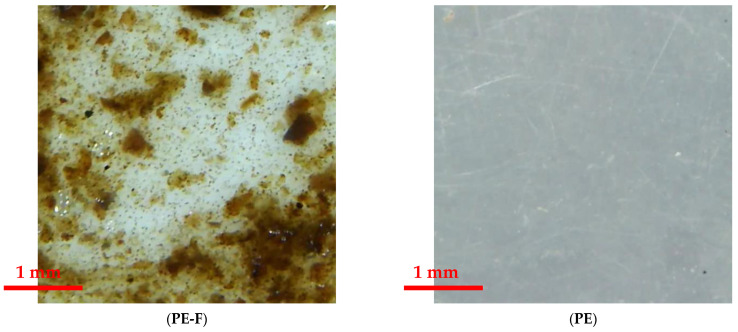
Microscopic photos of PE–pomace based membranes.

The results of microscopic measurements confirmed that the average size of the fraction of plant particles in the different types of membrane was similar (Table 5, Figure 6). Also, the percentage of plant particles per specific membrane area was convergent. On the basis of the presented results, it can be concluded that the size of the particles and their distribution in the membrane is not the factor determining the differences in the results of measurements of physical and mechanical properties of PE–pomace based membranes. Bearing the above in mind, it can be concluded that the reasons for the differences in the physical and mechanical parameters of the obtained membranes should rather be seen in the chemical composition of individual plant additives.

## 4. Conclusions

Food industry by-products can be a valuable source of raw material for the sustainable production of biomaterials for a variety of applications. In the presented work, PE–pomace based membranes were obtained, containing 50% of the plant component. Membranes were produced by melting and pressing under high pressure. The research analysis of the materials showed that they are characterized by low mechanical properties. At the same time, low water vapor permeability and wettability of membranes were noted, which means that the barrier properties of the tested materials are very good, which determines their potential use where such a feature is desired. Good barrier properties and low wettability are parameters especially desirable in food packaging, therefore the developed material could be a suitable and sustainable alternative to bioplastics based solely on synthetic polymers. Undoubtedly, one should strive to improve the mechanical properties of the produced membranes, either by improving the production method by optimizing the production process, optimizing the proportion of plant additives or by attempting to modify the plant raw material in order to increase the cohesion between molecules or to impart chemical reactivity.

Future research projects should aim at improving the qualitative characteristics of the obtained biomaterials, especially mechanical properties, and analyze the thermal stability and behavior in the environment to demonstrate that the developed biomaterials will be competitive to those already available on the market.

## Figures and Tables

**Table 1:** Measurement of maximum breaking load, Young’s modulus and thickness of PE–pomace based membranes.

Parameter	PE-B (SD) *	PE-F (SD) *	PE-C (SD) *	PE (SD) *
Breaking load [N]	16.03 ^a^ (3.88)	43.50 ^b^ (3.30)	46.10 ^b^ (6.07)	121.20 ^c^ (23.95)
Thickness [mm]	0.31 ^a^ (0.01)	0.42 ^b^ (0.01)	0.42 ^b^ (0.02)	0.31 ^a^ (0.02)
Young’s modulus [GPa]	12.63 ^a^ (0.95)	10.3 ^a^ (0.86)	18.27 ^b^ (1.17)	0.32 ^c^ (0.04)

* means and standard deviations in parentheses; ^abc^ is the homogeneous groups by the Tukey test (α = 0.05).

**Table 2:** Analysis of the variance of the influence of formulation of tested membranes on individual properties.

Parameter	Sum of Squares (SS)	Significance Level (p)	Percentage of Contribution (Pc)	Error *
Breaking load [N]	45,752.8	0.000000	92.2	7.8
Elongation [mm]	835,625.6	0.00	96.6	3.4
Thickness [mm]	0.076486	0.000000	84.1	15.9
Tensile strength [MPa]	737.9601	0.00	94.6	5.4
Young’s modulus	505.461	0.000000	98.3	1.7
WVP [g/m^2^ · 24 h]	61,517.8	0.022900	72.4	27.6
Contact angle [°] (5 s)	7291.9	0.00	91.2	8.8
Contact angle [°] (20 s)	8297.8	0.00	92.1	7.9
Contact angle [°] (40 s)	8531.2	0.00	92.6	7.4
Contact angle [°] (60 s)	8616.0	0.00	93.3	6.7
Shrinkage 300 s [%] Shrinkage 1800 s [%]	20.72165	0.000052 0.000135	74.5 71.2	25.5
	74.8326			28.8

* percentage of factor factors not accounted for in the study.

**Table 3:** Water vapor permeability (WVP) and shrinkage PE–pomace based membranes.

Parameter	PE-B (SD) *	PE-F (SD) *	PE-C (SD) *	PE (SD) *
WVP [g/m^2^ · 24 h]	256.80 ^b^	105.99 ^ab^	110.06 ^ab^	55.03 ^a^
Shrinkage 300 s [%]	0.47 ^a^ (0.32)	1.00 ^a^ (0.29)	1.33 ^a^ (1.12)	3.18 ^b^ (0.56)
Shrinkage 1800 s [%]	3.08 ^a^ (1.37)	3.08 ^a^ (1.37)	2.31 ^a^ (1.02)	7.23 ^b^ (1.66)

* means and standard deviations in parentheses; ^abc^ are the homogeneous groups by the Tukey test (α = 0.05).

**Table 4:** Measurement of surface wettability of PE–pomace based membranes.

Parameter	Time [s]	PE-B (SD) *	PE-F (SD) *	PE-C (SD) *	PE (SD) *
Contact angle [°] for water	5	35.3 a (5.3)	60.9 b (4.2)	69.7 c (4.5)	69.3 c (4.5)
	20	32.5 a (4.3)	60.7 b (4,4)	69.3 c (4.2)	68.5 c (4.7)
	40	31.7 a (4.2)	60.0 b (4.1)	69.2 c (4.2)	68.1 c (4.8)
	60	31.2 a (4.1)	59.1 b (3.8)	69.1 c (4.2)	67.7 c (4.9)

* means and standard deviations in parentheses; ^abc^ are the homogeneous groups by the Tukey test (α = 0.05).

**Table 5:** Measurement of pomace after oil extraction particles size and their percentage share in PE–pomace based membrane’s surface.

Parameter	PE-B (SD) *	PE-F (SD) *	PE-C (SD) *
Particle size range [µm]	0.148 × 0.227 (0.091)	0.152 × 0.226 (0.112)	0.160 × 0.270 (0.163)
Plant particles in 10 mm^2^ [%]	59.52 (2.54)	52.16 (6.87)	58.30 (3.09)

## Data Availability

Not applicable.
